# Xpert MTB/RIF assay for the diagnosis of rifampicin resistance in different regions: a meta-analysis

**DOI:** 10.1186/s12866-019-1516-5

**Published:** 2019-08-05

**Authors:** Kaican Zong, Chen Luo, Hui Zhou, Yangzhi Jiang, Shiying Li

**Affiliations:** 1 Department of Respiratory Medicine, The Seventh People’s Hospital of Chongqing, Chongqing, People’s Republic of China; 2 Department of Infectious Disease, The Second Affiliated Hospital, Chongqing Medical University, 74# Linjiang Road, Chongqing, 400010 People’s Republic of China

**Keywords:** Xpert MTB/RIF, Rifampicin resistance, Prevalence, Income, Meta-analysis

## Abstract

**Background:**

To estimate the diagnostic accuracy of Xpert MTB/RIF for rifampicin resistance in different regions, a meta-analysis was carried out.

**Methods:**

Several databases were searched for relevant studies up to March 3, 2019. A bivariate random-effects model was used to estimate the diagnostic accuracy.

**Results:**

We identified 97 studies involving 26,037 samples for the diagnosis of rifampicin resistance. The pooled sensitivity, specificity and AUC of Xpert MTB/RIF for rifampicin resistance detection were 0.93 (95% CI 0.90–0.95), 0.98 (95% CI 0.96–0.98) and 0.99 (95% CI 0.97–0.99), respectively. For different regions, the pooled sensitivity were 0.94(95% CI 0.89–0.97) and 0.92 (95% CI 0.88–0.94), the pooled specificity were 0.98 (95% CI 0.94–1.00) and 0.98 (95% CI 0.96–0.99), and the AUC were 0.99 (95% CI 0.98–1.00) and 0.99 (95% CI 0.97–0.99) in high and middle/low income countries, respectively. The pooled sensitivity were 0.91 (95% CI 0.87–0.94) and 0.91 (95% CI 0.86–0.94), the pooled specificity were 0.98 (95% CI 0.96–0.99) and 0.98 (95% CI 0.96–0.99), and the AUC were 0.98 (95% CI 0.97–0.99) and 0.99 (95% CI 0.97–0.99) in high TB burden and middle/low prevalence countries, respectively.

**Conclusions:**

The diagnostic accuracy of Xpert MTB/RIF for rifampicin resistance detection was excellent.

## Background

Tuberculosis (TB) remains a major global health problem and ranks as the leading cause of death from an infectious disease worldwide. In 2017, TB infected about 10.0 million people and approximately 16% (1.6 million) of infected patients died from the disease, which was a higher global total for new TB cases and deaths than previous one. Of the 1.6 million died cases, 300,000 occurred among people infected with human immunodeficiency virus (HIV) [1].

Drug-resistant TB, including multidrug-resistant TB (MDR-TB, defined as resistance to at least isoniazid and rifampicin, the two most important first-line anti-TB drugs) and extensively drug-resistant TB (XDR-TB, defined as MDR-TB plus resistance to any fluoroquinolone, such as ofloxacin or moxifloxacin, and to at least one of three injectable second-line drugs, amikacin, capreomycin, or kanamycin) has become a serious threat to global health [2]. In 2017, approximately 460,000 people, which means 3.5% of new and 18% of previously treated TB cases, were estimated to have had MDR-TB globally. And 9.0% of them had developed to XDR-TB. Rifampicin resistance (RR) was the most common resistance drug, affected approximately 558,000 people [1].

When TB is detected and effectively treated, the disease is largely curable. However, accurate and rapid detection of TB can be difficult, as challenging sample collection from deep-seated tissues and the paucibacillary characteristics of the disease [3]. Worldwide, approximately 35% of all forms of TB and 75% of patients with MDR-TB remain undiagnosed [4]. Notablely, under 3% of people who diagnosed with TB are tested to have certain pattern of drug resistance [5]. Xpert MTB/RIF was an effective, rapid, new method to diagnose TB and RR-TB, which was recommended by WHO [1].

Traditionally, the best available reference standard for TB diagnosis is solid and/or liquid culture. However, in clinical practice, prolonged turnaround times and limited laboratory infrastructure in resource-limited settings undermine the utility of culture-based diagnosis [6]. Histology is widely used for the diagnosis of TB where the technical pathologists are available However, it is time-consuming, technically demanding, and lacks specificity [7]. In early 2011, the World Health Organization (WHO) endorsed the Xpert® MTB/RIF assay (Cepheid, Sunnyvale, USA) [8], a novel, rapid, automated, cartridge-based nucleic acid amplification test (NAAT), for the initial diagnosis in patients with suspected pulmonary MDR-TB or HIV-associated pulmonary TB [9, 10]. It can simultaneously detect TB through detection of the DNA of *Mycobacterium tuberculosis* and simultaneously identify a majority of the mutations that confer rifampicin resistance (which is highly predictive of MDR-TB). A high accuracy for pulmonary TB detection (sensitivity 89%, specificity 99%) was obtained [11]. In late 2013, WHO expanded its recommendations to include the diagnosis of TB in children and some forms of extrapulmonary TB (EPTB) [1].

A series of meta-analyses were carried out to determine the diagnostic accuracy of Xpert MTB/RIF in different forms of TB [12–14], however, evaluation of its accuracy in rifampicin resistance is rare [11]. More importantly, no study estimated the diagnostic accuracy of Xpert MTB/RIF for rifampicin resistance in countries with different TB prevalence and income till now. To replenish this, in this review, we synthesized the available data, taking into account the accuracy of Xpert MTB/RIF in diagnosing rifampicin resistance.

## Methods

### Literature search strategy

We searched the MEDLINE, Cochrane library, EMBASE, and Web of Knowledge for published works without language restrictions. The key searching words were used were: “Xpert MTB/RIF”, “Xpert”, “Gene Xpert”, plus “rifampicin resistance”. Our last search was accomplished on March 3, 2019.

### Study selection and data extraction

The study selection and data extraction procedures were performed by two researchers (Kaican Zong and Hui Zhou) independently. Any differences in the process were solved by discussing with a third author (Shiying Li).

#### Inclusion criteria and exclusion criteria

Studies included in our meta-analysis should meet the following criteria: (i) clinical trials that used Xpert MTB/RIF for the detection of rifampicin resistance; (ii) samples were body tissues or fluid from suspected TB patients; (iii) the number of cases were more than 10; (iv) original data were sufficient to calculate the true positive (TP), true negative (TN), false positive (FP), and false negative (FN); (v) drug-susceptibility testing (DST) was used as the gold standard. Studies were excluded from our meta-analysis if they were: (i) case report; (ii) abstract of any conference; (iii) non-clinical research; (iv) review.

#### Data extraction

The following data were extracted from each included study: first author, year of publication, country, study settings, gender, the number of patients, the number and type of samples, diagnostic characteristics of Xpert MTB/RIF such as TP, TN, FP and FN. We sent e-mails to the authors for more details when data of individual studies were insufficient for a meta-analysis. In the case of inability to obtain data from the authors, the studies were excluded.

### Statistical analysis

MIDAS modules in the STATA statistical software (version 12.0; STATA Corporation, College Station, TX, USA) was used to perform the meta-analyses. The summary receiver operating characteristic (SROC) model and the bivariate random-effects model were used in our study to evaluate the diagnostic accuracy of Xpert MTB/RIF for rifampicin resistance detection. For each study, we calculated the sensitivity and specificity of Xpert MTB/RIF to diagnose rifampicin resistance along with 95% confidence intervals.

Quality Assessment of Diagnostic Accuracy Studies (QUADAS-2) tool was introduced to assess the quality of each included study. The Review Manager software (version 5.3, The Nordic Cochrane Centre, Copenhagen, Denmark) was used to present the result of QUADAS assessment.

We assessed the heterogeneity between included studies by using a bivariate boxplot, which can describe the degree of interdependence including the central location and identification of any outliers with an inner oval representing the median distribution of the data points and an outer oval representing the 95% confidence bound (by visually examining the position of each individual study, within the range of boxplot suggesting more heterogeneity).

## Results

### Description of included studies

**Fig. 1:**
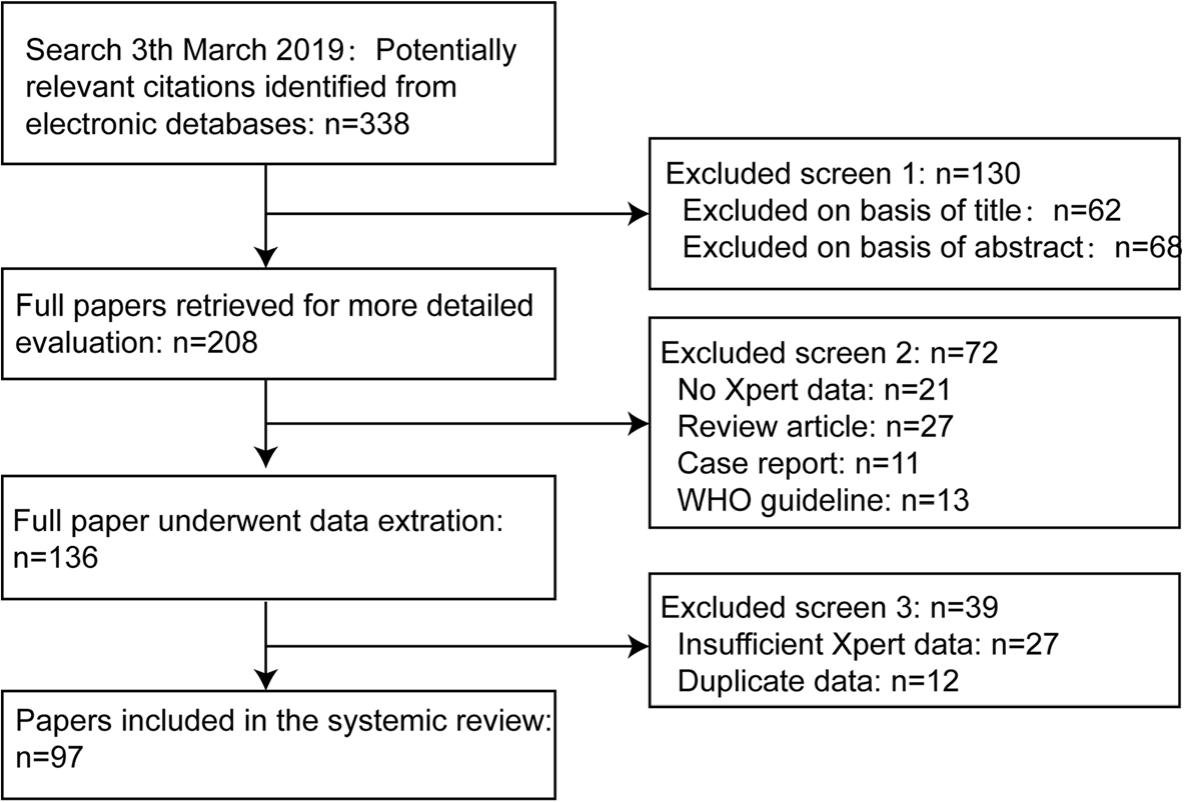
Flow diagram for literature search and selections of studies in this meta-analysis

Finally, we included 97 studies in this meta-analysis [15–111] (Fig. 1), including 26,037 samples for the diagnosis of rifampicin resistance. All studies were in English except five (three in Chinese [46, 64, 111] and two in Turkish [31, 79]). Twenty-six studies (26.8%) were conducted in high income countries (the World Bank income classification 2018) and 52 studies (53.6%) were in the 22 countries with a high burden of TB [1].

The median number of samples per study was 268 for rifampicin resistance detection. The samples of 56 included studies were pulmonary, such as sputum and BAL. Another 15 studies were extrapulmonary samples (e.g. body fluid, FNA, stool and blood), 16 studies included samples of both pulmonary and extrapulmonary (Tables 1 and 2).

**Table 1:** Characteristics of studies included in the meta-analysis for rifampicin-resistance tuberculosis detection

Study	First author [ref.]	Year	Country	Study setting	Male (%)	HIV (%)	Age (year) (Median, IQR)	Patient selecting method	Total samples n (included n)	Specimen type (samples n)	Gold standard
1	Al-Ateah SM [15]	2012	Saudi Arabia	Laboratory	126 (53.8)	1 (0.4)	NR	Cross-sectional Unspecified	234 (239)	Sputum (56), BAL (116); tissue (16), CSF (14), FNA (5), body fluid (22), abscess (10)	DST
2	Antonenka U [16]	2013	German	Clinical	NR	NR	NR	Retrospective Unspecified	121 (121)	Respiratory specimens (121)	Solid or liquid media DST
3	Balcells ME [17]	2012	Chile	Clinical	127 (79.4)	160 (100)	Adults> 18 (37.4, 19–65)	Cross-sectional Prospective Consecutive	160 (12)	Sputum (160)	Solid and liquid media DST
4	Barmankulova A [18]	2015	Kyrgyzstan	Laboratory	172 (57.3)	NR	Median 34, IQR 25–45	Cross-sectional Unspecified	300 (191)	Sputum (300)	Solid and liquid media DST
5	Barnard M [19]	2012	South Africa	Laboratory	NR	NR	NR	Unspecified Consecutive	282 (68)	Sputum (282)	DST
6	Bates M [20]	2013	Zambia	Clinical	NR	22 (2.4)	Children≤15	Prospective Unspecified	930 (930)	Sputum, gastric lavage aspirate (930)	Liquid culture
7	Biadglegne F [21]	2014	Ethiopia	Clinical	99 (42.9)	NR	14.7% ≤ 14, 85.3% > 14	Cross-sectional Unspecified	231 (32)	Lymph node aspirates (231)	DST
8	Blakemore R [22]	2010	America	Clinical	NR	NR	NR	Unspecified Unspecified	168 (79)	Sputum (168)	DST
9	Boehme CC [23]	2010		Clinical	929 (53.7)	392 (22.7)	Adults≥18 (34, 17–88)	Prospective Consecutive	1730 (720)	Sputum (1730)	Solid media DST
			Peru		181 (53.1)	3 (0.9)	Adults≥18 (31, 18–79)		341 (209)	Sputum (341)	Solid media DST
			Azerbaijan		251 (71.1)	9 (2.6)	Adults≥18 (37, 20–69)		353 (143)	Sputum (353)	Solid or liquid media DST
			South Africa		357 (49.2)	376 (51.8)	Adults≥18 (34, 18–74)		726 (183)	Sputum (726)	Liquid media DST
			India		140 (45.2)	4 (12.9)	Adults≥18 (30, 17–88)		310 (185)	Sputum (310)	Liquid media DST
10	Boehme CC [24]	2011		Clinical	4043 (60.8)	1255 (18.9)	Adults≥18 (38, 29–50)	Unspecified Consecutive	6648 (1060)	Sputum (6648)	DST
			Peru		607 (51.2)	5 (0.4)	Adults≥18 (37, 26–53)		1185 (185)	Sputum (1185)	Liquid media DST
			Azerbaijan		748 (99.9)	1 (0.1)	Adults≥18 (36, 30–44)		749 (211)	Sputum (749)	Liquid media DST
			South Africa		1275 (50.6)	947 (37.5)	Adults≥18 (36, 29–46)		2522 (188)	Sputum (2522)	Solid media DST
			Uganda		202 (54.3)	254 (68.3)	Adults≥18 (32, 26–38)		372 (116)	Sputum (372)	Solid media DST
			India		628 (69.6)	40 (4.4)	Adults≥18 (45, 32–58)		902 (103)	Sputum (902)	Solid media DST
			Philippines		583 (63.5)	8 (0.9)	Adults≥18 (47, 34–58)		918 (257)	Sputum (918)	DST
11	Bowles EC [25]	2011	Netherlands	Clinical	NR	NR	NR	Unspecified Unspecified	89 (60)	Sputum (86), pleural fluid (1), gastric fluid (1), bronchial washing (1)	DST
12	Carriquiry G [26]	2012	Peru	Clinical	95 (73)	131 (100)	Adults≥18 (35, 29–42)	Cross-sectional Unspecified	131 (39)	Sputum (131)	Solid and liquid media DST
13	Cayci YT [27]	2017	Turkey	Laboratory	NR	NR	NR	Unspecified Unspecified	34 (34)	Respiratory (19) and Non-respirator specimens (15)	Liquid media DST
14	Chakravorty S [28]	2017	South, Africa, India	Laboratory	NR	NR	NR	Prospective Unspecified	139 (139)	Sputum (139)	Liquid media DST
15	Chiang TY [29]	2018	China	Clinical	876 (29.6)	NR	Median 55, IQR 35.8–70.0	Prospective Unspecified	2957 (697)	Sputum (697)	Solid and liquid culture
16	Chikaonda T [30]	2017	Malawi	Clinical	NR	200 (57.0)	Adult≥18	Retrospective Random	351 (188)	Sputum (60)	Solid and liquid media DST
17	Ciftçi IH [31]	2011	Turkey	Clinical	NR	NR	NR	Unspecified Unspecified	85 (24)	Sputum (50), BAL (25), thorasynthesis fluid (5), urine (5)	Liquid media DST
18	Deggim V [32]	2013	Switzerland	Clinical	NR	NR	NR	Prospective Unspecified	79 (10)	Respiratory and Non-respirator specimens (79)	DST
19	Dharan NJ [33]	2016	Russia, Peru, Hong Kong, Haiti, USA	Clinical	358 (65.8)	536 (98.5)	Median 54.2, IQR 19–88	Unspecified, Unspecified	544 (185)	Sputum (185)	DST
20	Dorman SE [34]	2012	South Africa	Laboratory	6469 (93.8)	602 (8.7)	Median 43, IQR 34–49	Cross-sectional Consecutive	6893 (144)	Sputum (6893)	Liquid media DST
21	Dorman SE [35]	2018	South Africa, Uganda, Kenya, India, China, Georgia, Belarus, Brazil	Clinical	1059 (60.4)	441 (25.2)	Median 38, IQR 28–50	Prospective Unspecified	1753 (551)	Sputum (551)	Liquid media DST
22	Du J [36	2015	China	Clinical	70 (55.6)	5 (4.0)	Adults> 16 (38.6, 25.4–51.8)	Unspecified Unspecified	126 (126)	Pleural biopsy (126), pleural fluid specimens (126)	Liquid media DST
23	Feliciano CS [37]	2018	Brazil, Mozambique	Clinical	22 (75.9)	6 (20.7)	NR	Cross-sectional Unspecified	29 (29)	NR (29)	Solid media DST
24	Giang do C [38]	2015	Vietnam	Clinical	98 (65.3)	0 (0)	Children< 15 (18.5 months, 5–170 months)	Prospective Consecutive	150 (29)	Sputum (79), Gastric fluid (215), CSF (3), Pleural fluid (4), Cervical lymphadenopathic pus (1)	Liquid media DST
25	Gu Y [39]	2015	China	Clinical	28 (46.7)	NR	Median 39.7, IQR 19.5–74.6	Prospective Unspecified	60 (24)	Pus specimens (60)	Liquid media DST
26	Guenaoui K [40]	2016	France	Laboratory	35 (0.7)	NR	NR	Prospective Unspecified	50 (50)	Sputum (50)	Liquid DST
27	Helb D [41]	2010	Uganda	Clinical	38 (59.3)	20 (31.3)	Median 34, IQR 18–60	Retrospective Consecutive	64 (64)	Sputum (64)	DST
28	Hillemann D [42]	2011	German	Laboratory	NR	NR	NR	Unspecified Consecutive	521 (29)	Urine (91), gastric aspirate (30), tissue (245), pleural fluid (113), CSF (19), stool (23)	Liquid media DST
29	Huang H [43]	2018	China	Laboratory	NR	NR	NR	Retrospective Unspecified	2910 (1066)	NR	Liquid media DST
30	Huh HJ [44]	2014	South Korea	Clinical	197 (65.7)	1 (0.3)	Median 58, IQR 18–93	Retrospective Unspecified	300 (98)	Sputum (264), Bronchial washing or BAL (39)	Solid and liquid media DST
31	Hu P [45]	2014	China	Laboratory	1037 (76.7)	NR	3.2% < 20, 96.8% ≥ 20	Unspecified Consecutive	1352 (332)	Sputum (1352)	Solid media DST
32	Jin YH [46]	2017	China	Clinical	59 (54.1)	NR	Median 48.6, IQR 24.0–73.1	Unspecified Unspecified	109 (48)	Pus (48)	Liquid media DST
33	Kawkitinarong K [47]	2017	Thailand	Clinical	284 (58.6)	128 (25.9)	Median 41, IQR 30.8–54.3	Prospective Unspecified	521 (228)	Pulmonary specimens (228)	DST
34	Khalil KF [48]	2015	Pakistan	Clinical	36 (38.7)	0 (0)	> 16, 19.5–57.6	Unspecified Consecutive	93 (93)	BAL (93)	Solid media DST
35	Kim CH [49]	2014	South Korea	Clinical	104 (60.8)	1 (0.6)	Median 58.6, IQR 41.02–76.18	Retrospective Unspecified	171 (26)	Pulmonary (160), Non-pulmonary (38) specimens	Solid media DST
36	Kim CH [50]	2015	South Korea	Clinical	217 (56.7)	1 (0.3)	Median 56.31, IQR 38.43–74.18	Retrospective Convenience	383 (444)	Sputum (176), Bronchial washes (225), BAL (4); Pleural fluid (36), Tissue (1), Pericardial fluid (1), Lymph node (1)	Solid media DST
37	Kim MJ [51]	2015	South Korea	Laboratory	NR	NR	NR	Unspecified Convenience	52 (45)	Sputum (36), bronchial washing (10), pleural fluid (3), pleural mass (1), urine (2)	Liquid media DST
38	Kim SY [52]	2012	South Korea	Clinical	NR	NR	NR	Unspecified Consecutive	71 (62)	Sputum (71)	Solid and liquid DST
39	Kim YW [53]	2015	South Korea	Clinical	761 (53.3)	12 (0.8)	Median 59, IQR 0–99	Retrospective Consecutive	1429 (1540)	LN and tissue/pus (397), body fluid (469), CSF (254), joint fluid (283), urine (106), others (31)	Solid media DST
40	Kim YW [54]	2015	South Korea	Clinical	196 (61.1)	NR	Median 56, IQR 38–71	Retrospective Consecutive	321 (321)	Sputum (321)	DST
41	Kokuto H [55]	2015	Japan	Clinical	51 (54.8)	0 (0)	Adult≥20 (59.6, 45.0–75.0)	Retrospective Convenience	93 (56)	fecal specimens (93)	DST
42	Kostera J [56]	2018	Bangladesh	Clinical	NR	NR	NR	Unspecified Unspecified	132 (122)	Sputum (122)	Liquid media DST
43	Kurbaniyazova G [57]	2017	Kyrgyzstan	Laboratory	NR	NR	Adult≥18	Retrospective Unspecified	2734 (364) (414)	NR	Solid and liquid media DST
44	Kurbatova EV [58]	2013	Russia	Clinical	NR	NR	Adults≥18	Unspecified Consecutive	201 (99)	Sputum (201)	Solid and liquid media DST
45	Kwak N [59]	2013	South Korea	Clinical	426 (62.5)	5 (0.7)	Median 61, IQR 47.5–73.0	Retrospective Unspecified	681 (127)	Sputum (127)	Solid media DST
46	Lawn SD [60]	2011	South Africa	Clinical	162 (34.6)	468 (100)	Adults≥18 (33.6, 27.8–40.7)	Prospective Consecutive	468 (55)	Sputum (468)	Liquid media DST
47	Lee HY [61]	2013	South Korea	Clinical	78 (59.1)	1 (0.8)	Median 54.0, IQR 18–90	Retrospective Unspecified	132 (132)	Bronchoscopy specimens (132)	Ogawa media DST
48	Li Q [62]	2016	China	Laboratory	NR	NR	NR	Unspecified Consecutive	1973 (449)	Sputum (449)	Liquid media DST
49	Li Y [63]	2017	China	Laboratory	251 (60.6)	NR	Median 48.5, IQR 38.3–58.7	Unspecified Consecutive	420 (59)	Extra-pulmonary specimens (59)	Solid media DST
50	Liu X [64]	2015	China	Clinical	NR	NR	NR	Unspecified Unspecified	134 (44)	Pleural biopsy and pleural fluid specimens (100)	Liquid media DST
51	Lorent N [65]	2015	Cambodia	Clinical	160 (53.5)	189 (64.5)	Median 43, IQR 34–52	Prospective Consecutive	299 (102)	Sputum (102)	Solid media DST
52	Luetkemeyer AF [66]	2016	USA South Africa Brazil	Laboratory	446 (45.0)	617 (62.2)	Median 46, IQR 35–64	Unspecified Unspecified	992 (194)	Sputum (2)	DST
53	Metcalfe JZ [67]	2016	Zimbabwe	Clinical	216 (61.4)	238 (67.6)	Median 36.3, IQR 29.0–44.4	Prospective Consecutive	352 (161)	Sputum (161)	Solid and liquid media DST
54	Mokaddas E [68]	2015	Kuwait	Laboratory	NR	NR	NR	Unspecified Unspecified	452 (452)	Sputum (287), FNA (66), pus (58), pleural fluid (14), tissue (10), other sterile fluids (8), urine (5), CSF (2), stool (2).	Liquid media DST
55	Moon HW [69]	2015	South Korea	Clinical	NR	NR	NR	Unspecified Unspecified	100 (100)	Respiratory specimens (100)	DST
56	Moure R [70]	2011	Spain	Clinical	NR	NR	NR	Retrospective Unspecified	122 (85)	Sputum (92), BA (12), pulmonary biopsy (1); pleural fluid (4), gastric aspirate (5), urine (2), stool (1),cerebrospinal fluid (3), ascitic fluid (2), lymph node aspirate (1), skin biopsy (1), mammary abscess (1)	DST
57	Mwanza W [71]	2018	Zambia	Laboratory	NR	NR	NR	Unspecified Consecutive	1070 (24)	NR (24)	Liquid media DST
58	Myneedu VP [72]	2014	India	Laboratory	NR	NR	NR	Unspecified Unspecified	134 (88)	Sputum (134)	Liquid media DST
59	N’guessan K [73]	2014	Cote d’Ivoire	Clinical	91 (75.8)	NR	Median 34.2, IQR 24.1–44.3	Unspecified Unspecified	120 (29)	Sputum (120)	Liquid media DST
60	N’Guessan K [74]	2018	Côte d’Ivoire	Clinical	715 (65.3)	130 (12)	Median 33, IQR 18–80	Cross-sectional Consecutive	1095 (162)	Sputum (162)	Liquid media DST
61	Nikolayevskyy V [75]	2018	Ukraine	Clinical	2393 (68.8)	1265 (36.4)	Median 38.3, IQR 27–51.6	Retrospective Unspecified	3478 (3167)	Pulmonary specimens (3167)	Solid and liquid media DST
62	Nicol MP [76]	2011	South Africa	Clinical	250 (55.3)	108 (23.9)	Children≤15 (19.4 months, 11.1–46.2 months)	Prospective Consecutive	452 (77)	Sputum (452)	DST
63	O’Grady J [77]	2012	Zambia	Clinical	446 (50.6)	595 (67.5)	Adults> 15 (35, 28–43)	Prospective Unspecified	881 (96)	Sputum (881)	Liquid media DST
64	Ou X [78]	2014	China	Laboratory	1741 (70.9)	NR	NR	Unspecified Consecutive	2454 (616)	Sputum (2454)	Solid media DST
65	Ozkutuk N [79]	2014	Turkey	Laboratory	NR	NR	NR	Unspecified Unspecified	2639 (133)	Sputum (721), BAL (757), gastric fluid (94), endotracheal aspirates (30), transtracheal aspirate (9); urine (341), pleural fluid (232), tissue (176), CSF (111), abscesses (94), peritoneal fluid (42), pericardial fluid (18), joint fluid (7), other (7)	Liquid media DST
66	Pan X [80]	2018	China	Clinical	120 (63.2)	NR	Median 46.7, IQR 16–84	Prospective Unspecified	190 (62)	Sputum,BAL (62)	DST
67	Pang Y [81]	2014	China	Clinical	128	NR	Children< 14	Prospective Consecutive	211 (10)	Gastric lavage aspirates (211)	Liquid media DST
68	Park KS [82]	2013	South Korea	Clinical	NR	NR	NR	Prospective Consecutive	320 (19)	Respiratory specimens (320)	Liquid media DST
69	Pimkina E [83]	2015	Lithuania	Laboratory	559 (70.6)	NR	Age ≥ 15	Retrospective Unspecified	791 (264)	Respiratory specimens (264)	Solid or liquid media DST
70	Pinyopornpanish K [84]	2015	Thailand	Clinical	34 (59.6)	15 (26.3)	≥15 (55.6, 35.5–75.7)	Cross-sectional Consecutive	57 (43)	Sputum(57)	Liquid media DST
71	Rachow A [85]	2011	Tanzania	Clinical	141 (48.3)	172 (58.9)	Median 39.2	Unspecified Consecutive	292 (61)	Sputum (292)	Liquid media DST
72	Rahman A [86]	2016	Bangladesh	Clinical	NR	NR	NR	Unspecified Unspecified	92 (92)	Sputum (92)	Liquid media DST
73	Raizada N [87]	2014	India	Clinical	2339 (50.8)	NR	Children< 14	Prospective Consecutive	4600 (48)	Sputum (4600)	DST
74	Reither K [88]	2015	Tanzania Uganda	Clinical	219 (45.6)	197 (43.7)	Children< 16 (5.6, 2.0–9.8)	Prospective Consecutive	451 (25)	Sputum (451)	Liquid media DST
75	Rice JP [89]	2017	America	Laboratory	NR	NR	Median 50, IQR 35–60	Retrospective Unspecified	637 (120)	Sputum (120)	Liquid media DST
76	Sharma SK [90]	2015	India	Laboratory	909 (64.7)	NR	Median 37.5, IQR 19.4–55.6	Unspecified Consecutive	1406 (422)	Respiratory specimens (422)	Solid and liquid media DST
77	Sharma SK [91]	2017	India	Laboratory	1405 (55.6)	NR	Median 35.29, IQR 20–50	Unspecified Convenient	2468 (328)	Extra-pulmonary specimens (328)	Liquid media DST
78	Singh UB [92]	2016	India	Clinical	589 (51.4)	NR	NR	Prospective Unspecified	1145 (72)	Pulmonary and Extra-pulmonary specimens (132)	Liquid media DST
79	Soeroto AY [93]	2019	Indonesia	Clinical	193 (56.9)	5 (1.5)	Median 38.2, IQR 25.7–50.7	Retrospective Unspecified	339 (158)	NR (158)	DST
80	Ssengooba W [94]	2014	Uganda	Clinical	155 (36.6)	424 (100)	Median 32, IQR 32–34	Prospective Unspecified	424 (9)	Sputum (424)	Liquid media DST
81	Strydom K [95]	2015	South Africa	Laboratory	NR	NR	NR	Retrospective Consecutive	120 (115)	Sputum (120)	Liquid media DST
82	Tahseen S [96]	2016	Pakistan	Clinical	1078 (54.3)	NR	Median 33	Cross-sectional Consecutive	1984 (1533)	Sputum (1533)	Solid media DST
83	Theron G [97]	2011	South Africa	Clinical	325 (67.7)	130 (27.1)	Adults≥18 (36, 18–83)	Unspecified Consecutive	480 (157)	Sputum (480)	Liquid media DST
84	Tsuyuguchi K [98]	2017	Japan	Clinical	146 (61.6)	NR	Median 65.2, IQR 23–94	Prospective Consecutive	237 (201)	Sputum (201)	Solid media DST
85	Ullah I [99]	2017	Pakistan	Clinical	130 (48.9)	0 (0)	Median 34, IQR 3–80	Unspecified Unspecified	266 (88)	Extra-pulmonary specimens (88)	DST
86	Vadwai V [100]	2011	India	Clinical	251 (45.9)	16 (2.9)	Median 37, IQR 8 months-94	Unspecified Consecutive	547 (125)	Biopsy (284), pus (147), body fluids (93), CSF (23)	Solid and liquid media DST
87	van Kampen SC [101]	2015	Kazakhstan	Laboratory	NR	52(0.9)	NR	Prospective Consecutive	5611 (1054)	Sputum (5611)	Solid or liquid media DST
88	van Kampen SC [102]	2015	Indonesia	Clinical	872 (60.5), missing 15(1.0)	35 (2.4)	0.5% < 15, 97.7% ≥ 16, 1.8% missing	Unspecified Consecutive	1442 (339)	Sputum (1442)	DST
89	Wang G [103]	2017	China	Clinical	NR	NR	NR	Prospective Undefined	1461 (538)	Pulmonary specimens (1063), extra-pulmonary specimens (398)	Solid media DST
90	Wang G [104]	2019	China	Clinical	192 (65.75)	0 (0)	Median 42, IQR 14–89	Prospective Consecutive	292 (119)	Sputum (90), pleural fluid (29)	Solid or liquid media DST
91	Williamson DA [105]	2012	New Zealand	Clinical	NR	NR	NR	Unspecified Unspecified	169 (14)	Respiratory specimens (89); extra-pulmonary specimens (9), MGIT liquid culture vials (71)	Liquid media DST
92	Yin QQ [106]	2014	China	Clinical	141 (55.3)	NR	Children≤18 (6.1, 0.3–15.3)	Unspecified Unspecified	255 (21)	BALF (255)	Liquid media DST
93	Yuan M [107]	2016	China	Clinical	NR	0 (0)	NR	Retrospective Unspecified	328 (90)	Extra-pulmonary specimens (90)	DST
94	Zar HJ [108]	2012	South Africa	Clinical	294 (55.0)	117 (21.9)	Children< 15 (19.0 months, 11.2–38.3 months)	Unspecified Consecutive	535 (125)	Nasopharyngeal specimens, sputum (535)	Liquid culture
95	Zar HJ [109]	2014	South Africa	Clinical	181 (47)	31 (8)	Children< 15 (38.3 months, 21.2–56.5 months)	Prospective Consecutive	384 (18)	Sputum (309), Nasopharyngeal aspirate specimens (309)	DST
96	Zetola NM [110]	2014	Botswana	Clinical	221 (59.7)	279 (59.4)	Adult≥18 (37, 31–44)	Retrospective Consecutive	370 (370)	Sputum (370)	DST
97	Zhang AM [111]	2016	China	Clinical	65 (59.6)	0 (0)	Children≤14	Unspecified Unspecified	109 (21)	Pulmonary and Extra-pulmonary specimens (21)	Liquid media DST

Sample selection: Study units selected prospectively, or retrospectively from existing samples; Consecutive, random or convenience sampling method. ‘Unspecified’ refers to studies where there was no clear indication how the study participants were chosen. Solid media culture(Löwensten-Jensen), liquid media culture (Bactec MGIT 960)

**Table 2:** Data of diagnostic accuracy of studies included in the meta-analysis for rifampicin resistance tuberculosis detection

Study	First author [ref.]	Year	Total samples n (included n)	True positive	False positive	False negative	True negative	Specimen type
Study	Al-Ateah SM [15]	2012	234 (59)	2	0	0	57	Respiratory and non-respiratory specimens
1	Antonenka U [16]	2013	121 (50)	2	0	0	48	Respiratory specimens
2	Balcells ME [17]	2012	160 (12)	0	2	0	10	Sputum
3	Barmankulova A [18]	2015	300 (191)	91	8	3	89	Sputum
4	Barnard M [19]	2012	282 (36)	3	0	0	33	Sputum
5	Bates M [20]	2013	930 (41)	2	1	0	38	Sputum, gastric lavage aspirate
6	Biadglegne F [21]	2014	231 (32)	2	1	0	29	Lymph node aspirates
7	Blakemore R [22]	2010	168 (79)	37	0	0	42	Sputum
8	Boehme CC [23]	2010	1730 (720)	200	10	5	505	Sputum
9		Peru	341 (209)	16	3	0	190	
		Azerbaijan	353 (143)	47	4	2	90	
		South Africa	726 (183)	18	0	1	164	
		India	310 (185)	119	3	2	61	
	Boehme CC [24]	2011	6648 (1060)	236	14	14	796	Sputum
10		Peru	1185 (185)	22	1	1	161	
		Azerbaijan	749 (211)	47	1	3	160	
		South Africa	2522 (188)	9	3	1	175	
		Uganda	372 (116)	1	1	2	112	
		India	902 (103)	8	2	2	91	
		Philippines	918 (257)	149	6	5	97	
	Bowles EC [25]	2011	89 (60)	8	0	0	52	Sputum, pleural fluid, gastric fluid, bronchial washing
11	Carriquiry G [26]	2012	131 (39)	6	3	0	30	Sputum
12	Cayci YT [27]	2017	34 (34)	3	1	0	30	Respiratory and none-respiratory specimens
13	Chakravorty S [28]	2017	139 (139)	38	1	3	97	Sputum
14	Chiang TY [29]	2018	2957 (697)	36	9	0	652	Sputum
15	Chikaonda T [30]	2017	351(200)	2	1	0	185	Sputum
16	Ciftçi IH [31]	2011	85 (24)	0	0	0	24	Sputum, BAL, thorasynthesis fluid, urine
17	Deggim V [32]	2013	79 (10)	0	3	0	7	Respiratory and None-respiratory
18	Dharan NJ [33]	2016	544 (185)	85	9	2	89	Sputum
19	Dorman SE [34]	2012	6893 (144)	5	5	0	134	Sputum
20	Dorman SE [35]	2018	1753 (551)	167	7	8	369	Sputum
21	Du J [36]	2015	126 (43)	9	2	1	31	Pleural biopsy specimen
22	Feliciano CS [37]	2018	29 (29)	12	3	4	10	NR
23	Giang do C [38]	2015	150(29)	1	0	0	28	Respiratory and non-respiratory specimens
24	Gu Y [39]	2015	60 (24)	6	0	0	18	Pus specimens
25	Guenaoui K [40]	2016	50 (50)	21	0	0	29	Sputum
26	Helb D [41] Uganda	2010	64 (64)	9	1	0	54	Sputum
27	Hillemann D [42]	2011	521 (29)	0	4	0	25	Non-respiratory specimens
28	Huang H [43]	2018	2910 (1066)	147	16	5	898	NR
29	Huh HJ [44]	2014	300 (98)	6	1	1	90	Respiratory specimens
30	Hu P [45]	2014	1352 (332)	26	4	2	300	Sputum
31	Jin YH [46]	2017	109 (48)	4	4	1	39	Pus
32	Kawkitinarong K [47]	2017	521 (228)	15	0	1	212	Pulmonary specimens
33	Khalil KF [48]	2015	93 (93)	5	0	1	87	BAL
34	Kim CH [49]	2014	171 (26)	2	0	0	24	Respiratory and non-respiratory specimens
35	Kim CH [50]	2015	383 (36)	4	1	0	31	Respiratory and Non Respiratory specimens
36	Kim MJ [51]	2015	52 (45)	1	0	1	43	Respiratory and non-respiratory specimens
37	Kim SY [52]	2012	71 (62)	21	0	0	41	Sputum
38	Kim YW [53]	2015	1429 (47)	4	0	1	42	Non-respiratory specimens
39	Kim YW [54]	2015	321 (321)	25	4	0	292	Sputum
40	Kokuto H [55]	2015	93 (56)	4	0	2	50	Fecal specimens
41	Kostera J [56]	2018	132 (122)	28	0	4	90	Sputum
42	Kurbaniyazova G [57]	2017	2734 (364, solid media DST)	120	20	12	212	NR
			2734 (414, liquid media DST)	108	29	13	264	NR
43	Kurbatova EV [58]	2013	201 (99)	57	1	5	36	Sputum
44	Kwak N [59]	2013	681 (127)	8	6	0	113	Sputum
45	Lawn SD [60]	2011	468 (55)	4	3	0	48	Sputum
46	Lee HY [61]	2013	132 (35)	2	0	0	33	Bronchoscopy specimens
47	Li Q [62]	2016	1973 (449)	47	16	6	380	Sputum
48	Li Y [63]	2017	420 (59)	11	0	1	47	Extra-pulmonary specimens
49	Liu X [64]	2015	134 (44)	10	2	1	31	Pleural biopsy and pleural fluid specimens
50	Lorent N [65]	2015	299 (102)	24	6	3	69	Sputum
51	Luetkemeyer AF [66]	2016	992 (194)	5	1	2	186	Sputum
52	Metcalfe JZ [67]	2016	352 (161)	54	8	9	90	Sputum
53	Mokaddas E [68]	2015	452 (452)	10	2	0	440	Respiratory and non-respiratory specimens
			smear(+)(179)	4	0	0	175	
			smear(−)(273)	6	2	0	265	
			pulmonary(287)	7	1	0	279	
			extrapulmonary(165)	3	1	0	161	
54	Moon HW [69]	2015	100 (100)	47	0	3	50	Respiratory specimens
55	Muñoz L [70]	2011	122 (85)	6	0	1	78	Respiratory and non-respiratory specimens
56	Mwanza W [71]	2018	1070 (24)	13	3	0	8	NR
57	Myneedu VP [72]	2014	134 (88)	54	1	1	32	Sputum
58	N’guessan K [73]	2014	120 (29)	14	4	0	11	Sputum
59	N’Guessan K [74]	2018	1095 (162)	112	8	0	42	Sputum
60	Nikolayevskyy V [75]	2018	3478 (3167)	1212	77	86	1792	Pulmonary specimens
61	Nicol MP [76]	2011	452 (77)	3	4	0	70	Sputum
62	O’Grady J [77]	2012	881 (96)	13	2	3	78	Sputum
63	Ou X [78]	2014	2454 (616)	54	16	8	538	Sputum
64	Ozkutuk N [79]	2014	2639 (133)	1	1	0	131	Respiratory and non-respiratory specimens
65	Pan X [80]	2018	190 (62)	2	2	0	58	Sputum and BAL
66	Pang Y [81]	2014	211 (10)	1	0	0	9	Gastric lavage aspirates
67	Park KS [82]	2013	320 (19)	2	0	0	17	Respiratory specimens
68	Pimkina E [83]	2015	791 (264)	39	4	0	221	Sputum
69	Pinyopornpanish K [84]	2015	57 (43)	0	0	3	40	Sputum
70	Rachow A [85]	2011	292 (61)	0	0	0	61	Sputum
71	Rahman A [86]	2016	92 (92)	85	6	0	1	Sputum
72	Raizada N [87]	2014	4600 (48)	47	1	0	0	Sputum
73	Reither K [88]	2015	451 (25)	0	0	0	25	Sputum
74	Rice JP [89]	2017	637 (120)	2	2	0	116	Sputum
75	Sharma SK [90]	2015	1406 (422)	104	7	6	305	Respiratory specimens
76	Sharma SK [91]	2017	2468 (328)	38	2	3	285	Extra-pulmonary specimens
77	Singh UB [92]	2016	1145 (72)	14	0	2	56	Pulmonary and extra-pulmonary specimens
78	Soeroto AY [93]	2019	339 (158)	141	17	0	0	NR
79	Ssengooba W [94]	2014	424 (94)	4	0	0	9	Sputum
80	Strydom K [95]	2015	120 (115)	59	1	2	53	Sputum
81	Tahseen S [96]	2016	1984 (1533)	85	17	15	1416	Sputum
82	Theron G [97]	2011	480 (157)	5	1	0	151	Sputum
83	Tsuyuguchi K [98]	2017	237 (201)	22	3	0	176	Sputum
84	Ullah I [99]	2017	266 (88)	24	2	0	62	Extra-pulmonary specimens
85	Vadwai V [100]	2011	547 (125)	39	5	1	80	Non-respiratory specimens
86	van Kampen SC [101]	2015	5611(1054)	522	31	33	468	Sputum
87	van Kampen SC [102]	2015	1442 (339)	158	18	21	142	Sputum
88	Wang G [103]	2017	1461 (538)	145	0	3	390	Pulmonary and extra-pulmonary specimens
89	Wang G 104]	2019	229 (119)	21	0	1	97	Sputum, pleural fluid
			90	15	0	1	74	Sputum
			29	6	0	0	23	Pleural fluid
90	Williamson DA [105]	2012	169 (14)	7	6	0	1	Respiratory; extra-pulmonary specimens, positive MGIT liquid culture vials
91	Yin QQ [106]	2014	255 (21)	1	0	0	20	BALF
92	Yuan M [107]	2016	328(90)	12	0	3	75	Extra-pulmonary specimens
93	Zar HJ [108]	2012	535 (125)	5	5	1	114	Nasopharyngeal specimens, sputum
94	Zar HJ [109]	2014	384 (18)	0	0	0	18	Sputum Nasopharyngeal aspirate specimens
95	Zetola NM [110]	2014	370 (370)	51	1	4	314	Sputum
97	Zhang AM [111]	2016	109 (21)	6	0	0	15	Pulmonary and extra-pulmonary specimens

*IQR* Interquartile range, *TA* Tracheal aspirate, *BA* Bronchial aspirate, *BAL* Bronchoalveolar lavage, *LN* Lymph node, *CSF* Cerebrospinal fluid, *EPTB* Extra-pulmonary tuberculosis, *CCRS* Composite clinical reference standard, *FNA* Fine needle aspirate; DST: drug-susceptibility testing

### Methodological quality of included studies

**Fig. 2:**
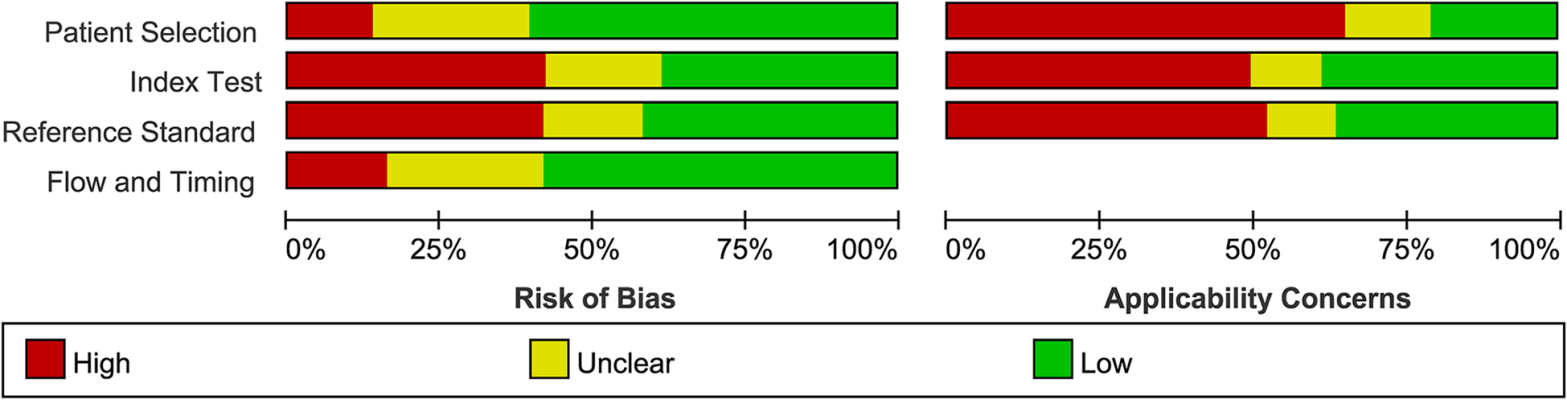
Risk of bias and applicability concerns as percentages across the included studies for rifampicin resistance detection

The overall methodological quality of the included studies was summarized in Fig. 2. Approximately half of the included studies collected data consecutively (*n* = 41; 42.2%) (Table 1) and no study used a case-control design. All studies were carried out either in tertiary care centers or reference laboratories. In index tests part, 15 studies (15.5%) were considered as unclear risk of bias. In reference standard part, 11 studies (11.3%) were considered as unclear risk of bias because the results of the reference standard were interpreted with unclear blind of the results of the index tests. In flow and timing part, 14 studies (24.7%) were considered as unclear risk of bias because not all patients were included in the analysis.

**Fig. 3:**
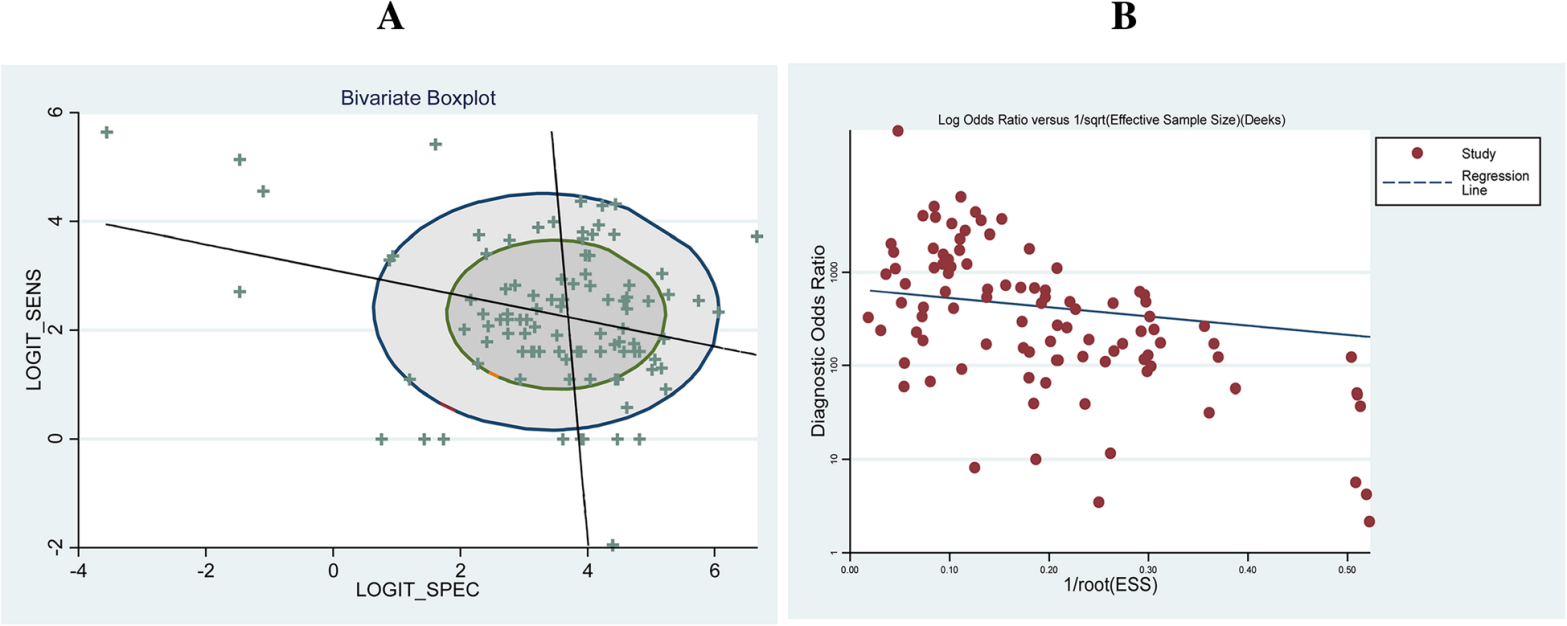
Heterogeneity test of included studies in this meta-analysis: a bivariate boxplot (**a**) and a Deek’s funnel plot (**b**)

The heterogeneity of the studies included in this study was tested by a bivariate boxplot (Fig. 3a) and a Deek’s funnel plot (Fig. 3b). Most of the included studies were in the bivariate boxplot, and the slope of Deek’s funnel was almost horizontal, which all meant a good heterogeneity.

### Detection of rifampicin resistance in different prevalence and income regions

**Fig. 4:**
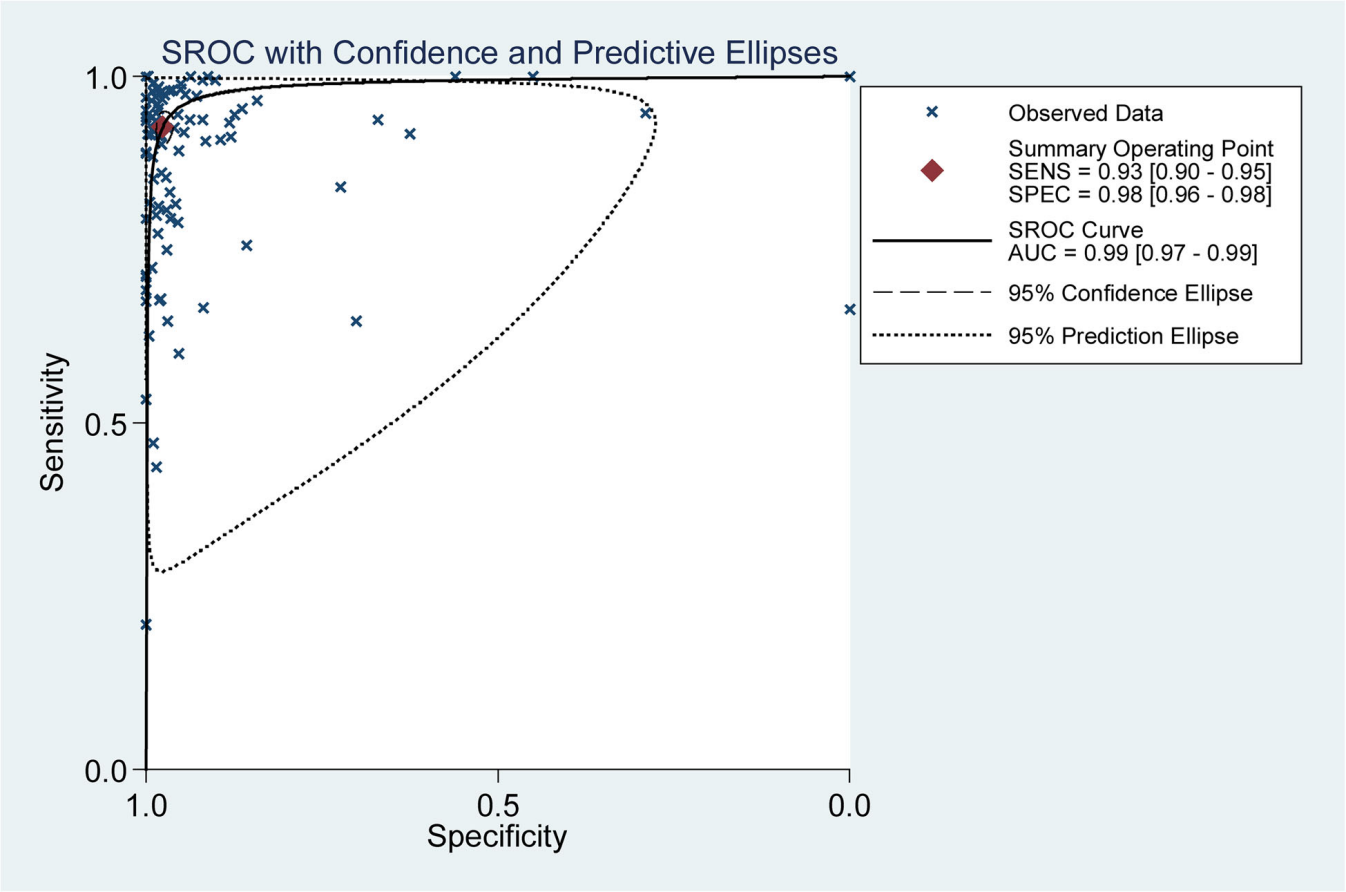
The SROC plot of Xpert MTB/RIF sensitivity and specificity for rifampicin resistance detection. The points represent the sensitivity and specificity of one study; the summary point represents the summary sensitivity and specificity

The accuracy of Xpert MTB/RIF for rifampicin resistance detection was estimated in 59 studies. The pooled sensitivity, specificity and AUC of Xpert MTB/RIF for detecting rifampicin resistance were 0.93 (95% CI 0.90–0.95), 0.98 (95% CI 0.96–0.98) and 0.99 (95% CI 0.97–0.99), respectively (Fig. 4).

**Fig. 5:**
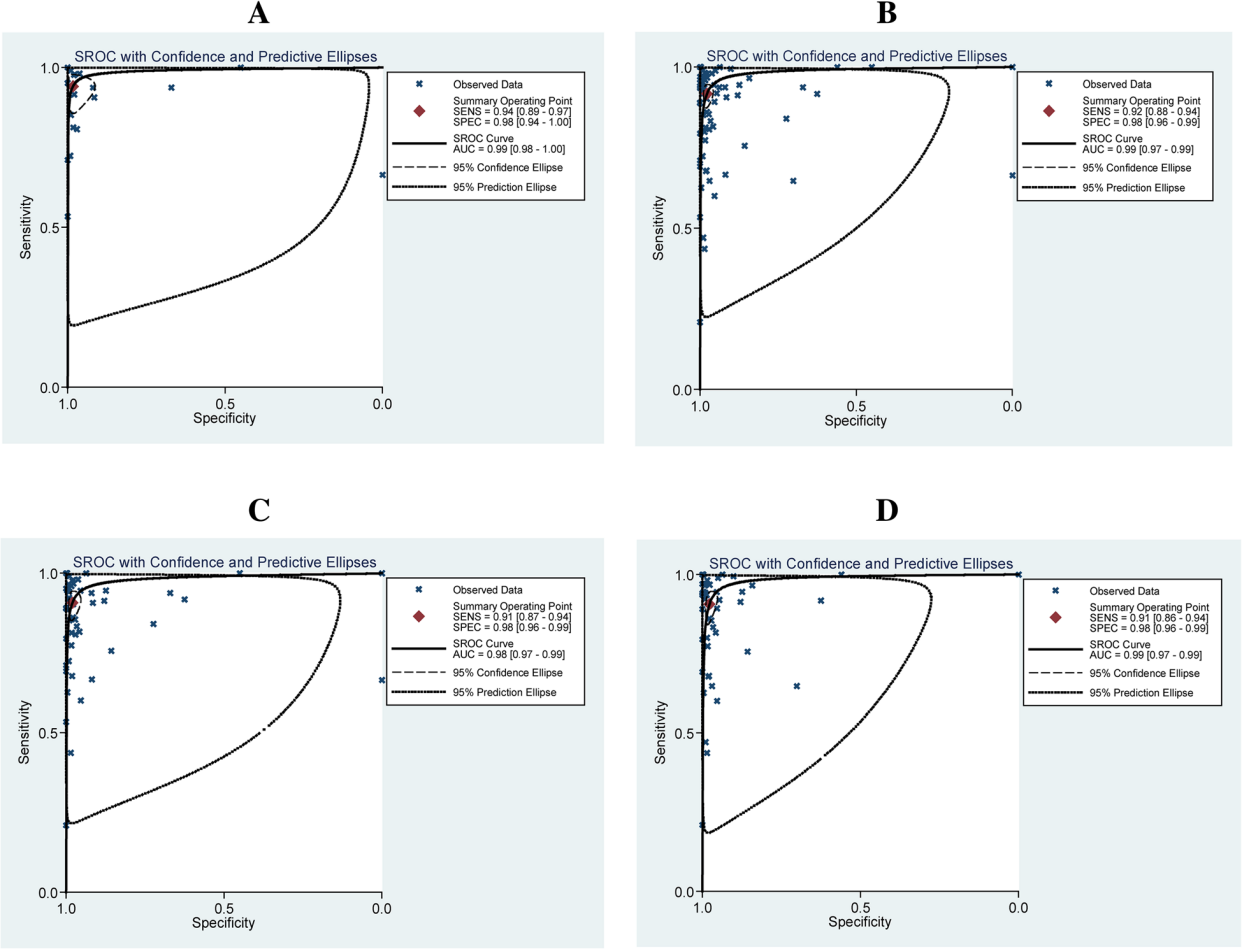
The SROC plot of Xpert MTB/RIF sensitivity and specificity for rifampicin resistance detection. **a** High income countries, **b** Middle/low income countries, **c** High TB burden countries, **d** Middle/low TB prevalence countries. The points represent the sensitivity and specificity of one study; the summary point represents the summary sensitivity and specificity

Of the 97 studies, 26 studies were of high income countries, 62 of middle and 9 were of low income. For TB prevalence, 52 studies were from the 22 high TB burden countries, and 45 were not. The pooled sensitivity were 0.94(95% CI 0.89–0.97) and 0.92 (95% CI 0.88–0.94), the pooled specificity were 0.98 (95% CI 0.94–1.00) and 0.98 (95% CI 0.96–0.99), and the AUC were 0.99 (95% CI 0.98–1.00) and 0.99 (95% CI 0.97–0.99) in high and middle/low income countries, respectively (Fig. 5a and Fig. 5b). The pooled sensitivity were 0.91 (95% CI 0.87–0.94) and 0.91 (95% CI 0.86–0.94), the pooled specificity were 0.98 (95% CI 0.96–0.99) and 0.98 (95% CI 0.96–0.99), and the AUC were 0.98 (95% CI 0.97–0.99) and 0.99 (95% CI 0.97–0.99) in high TB burden and middle/low prevalence countries, respectively (Fig. 5c and Fig. 5d).

## Discussion

Several meta-analyses have focused on the diagnostic accuracy of Xpert MTB/RIF for pulmonary [12] or extra-pulmonary TB [13, 14] detection either on adults or children [12]. However, to our knowledge, this is the first meta-analysis for Xpert MTB/RIF diagnostic accuracy for rifampicin resistance detection in different prevalence and income regions. Our systematic review demonstrated that Xpert MTB/RIF is high sensitive diagnostic tool for rifampicin resistance detection. Firstly, the accuracy of Xpert MTB/RIF for rifampicin resistance detection was estimated in our meta-analysis. As shown in Fig. 4, the accuracy of Xpert MTB/RIF for rifampicin resistance detection was impressive. The pooled sensitivity, specificity and AUC were 0.93 (95% CI 0.90–0.95), 0.98 (95% CI 0.96–0.98) and 0.99 (95% CI 0.97–0.99), respectively. As estimated, about 75% of multi-drug resistant TB remains undiagnosed [4]. We strongly hope Xpert MTB/RIF, which provided a quick and accurate result, will contribute to early and accurate diagnosis of rifampicin resistance.

The overall sensitivity of Xpert MTB/RIF for rifampicin resistance detection were almost the same between high TB prevalence countries and middle/low ones (0.91, 95% CI 0.87–0.94 versus 0.91, 95% CI 0.86–0.94). And for different income levels, the sensitivities of high income ones was also similar with the ones of middle/low income (0.94, 95% CI 0.89–0.97 versus 0.92, 95% CI 0.88–0.94). We can see, taking the different levels of TB prevalence and country income into account, no significant differences were found between subgroups, either in sensitivities, specificities and AUCs.

TB remains one of the world’s deadliest communicable diseases. However, it is intensively distributed in several high burden countries. In 2017, more than half of the new TB was developed in the South-East Asia and Western Pacific Regions. To be specific, one quarter were in the African Region. India and China alone accounted for 24 and 13% of the total cases, respectively [4]. Interestingly, the tendency of TB prevalence was consisted with the economic development at some degree. The income levels of the 22 high TB burden countries all were all middle or low, except one (Russian) [4]. Therefore, it is of significant meanings to estimate the diagnostic accuracy of Xpert MTB/RIF in countries with different levels of TB prevalence and income. Some researchers discovered that the Xpert MTB/RIF showed a higher sensitivity of TB detection in lower TB prevalence countries, which could significantly help the physicians to make clinical decisions [112]. However, our result, from another aspect, showed the diagnostic accuracy of Xpert MTB/RIF for rifampicin resistance detection was not differed between countries with different TB prevalence and incomes.

Advantages of this review were the use of a standard protocol, a bivariate random-effects model used for meta-analysis, and independent reviewers. The data set involved comprehensive searching to identify studies as well as repeated correspondence with authors of study to obtain additional data on the studies.

While there were still some limitations in our analysis. We may have missed some studies despite the comprehensive search. Secondly, sample processing was highly variable across and within studies, as there was no recommendation available on how to process non-respiratory samples from the manufacturer or the WHO.

## Conclusions

In conclusion, based on our meta-analysis, the diagnostic accuracy of Xpert MTB/RIF for rifampicin resistance detection was excellent. The overall sensitivity of Xpert MTB/RIF for rifampicin resistance detection in different TB prevalence and income countries were not significant different. We believe that the information obtained from this study will aid the decision making of physicians who take care of patients with possible resistant tuberculosis infection.

## Data Availability

The datasets used and/or analysed during the current study are available from the corresponding author on reasonable request.

## Abbreviations

BA: Bronchial aspirate
BAL: Bronchoalveolar lavage
CCRS: Composite clinical reference standard
CSF: Cerebrospinal fluid
DST: Drug-susceptibility testing
EPTB: Extra-pulmonary tuberculosis
FN: False negative
FNA: Fine needle aspirate
FP: False positive
HIV: Human immunodeficiency virus
IQR: Interquartile range
LN: Lymph node
MDR-TB: multidrug-resistant TB
NAAT: Nucleic acid amplification test
QUADAS: Quality assessment of diagnostic accuracy studies
RR: Rifampicin resistance
SROC: Summary receiver operating characteristic
TA: Tracheal aspirate
TB: Tuberculosis
TN: True negative
TP: True positive
WHO: World Health Organization
XDR-TB: Extensively drug-resistant TB
